# Early clinical and laboratory markers associated with post-COVID respiratory syndrome: A retrospective analysis

**DOI:** 10.1371/journal.pone.0344371

**Published:** 2026-03-13

**Authors:** Yulia Ariani, Indra Gunawan, Agus Dwi Susanto, Riyadi Sutarto

**Affiliations:** 1 Department of Medical Biology, Faculty of Medicine, University of Indonesia, Salemba, Jakarta, Indonesia; 2 Persahabatan National Respiratory Hospital, Rawamangun, Jakarta, Indonesia; 3 Department of Pulmonology and Respiratory Medicine, Faculty of Medicine, University of Indonesia, Salemba, Jakarta, Indonesia; Azienda Ospedaliero Universitaria Careggi, ITALY

## Abstract

**Background:**

Post-COVID-19 respiratory syndrome remains a significant concern, yet early clinical and laboratory markers at the time of admission are not well established. Identifying laboratory markers associated with this condition could help guide clinical management and long-term monitoring. This study aimed to determine which laboratory findings at admission significantly differ between COVID-19 survivors with and without post-COVID respiratory syndrome (PCRS) and assess their potential as markers.

**Methods:**

A retrospective comparative study was conducted on COVID-19 survivors who has history of hospitalization at Persahabatan National Referral General Hospital, Jakarta, in 2020–2021, divided into case (PCRS, n:43) and control (nonPCRS, n:42) groups. Demographic data, vital signs, and laboratory findings were analyzed, including complete blood count, kidney and liver function, electrolytes, blood gas analysis, D-dimer, and C-reactive protein (CRP).

**Results:**

Compared with controls, cases demonstrated significantly higher neutrophil percentages, neutrophil-to-lymphocyte ratio (NLR), blood urea nitrogen (BUN), potassium levels, and respiratory rates, along with lower lymphocyte and eosinophil percentages at admission. After Benjamini–Hochberg correction for multiple testing, respiratory rate, potassium, BUN, and neutrophil percentage remained statistically significant. In adjusted multivariable logistic regression models controlling for age, sex, body mass index, and markers of disease severity (SpO₂ and/or respiratory rate), potassium and respiratory rate showed consistent independent associations with case status across several models, while NLR retained a modest association only in models incorporating SpO₂. No significant differences were observed for D-dimer or CRP.

**Conclusion:**

Neutrophilia, lymphopenia, increased NLR, elevated BUN, potassium levels, and higher respiratory rates at admission were associated with post-COVID respiratory syndrome. Among these, potassium levels and respiratory rate showed more consistent associations after adjustment for demographic factors and disease severity markers. These findings describe admission characteristics linked to post-COVID-19 Respiratory syndrome. Larger prospective studies with serial measurements are needed to confirm their clinical relevance and prognostic value.

## Introduction

Long COVID is a chronic condition that occurs after SARS-COV 2 infection, where a person can show manifestations of multiorgan symptoms that are often severe and can last up to 4 weeks, 3 months, or even years [1,2]. It is estimated that long-COVID is a condition experienced by 10% of people infected with SARS-COV 2 [1]. Risk factors include female gender, higher BMI, experiencing severe covid symptoms, comorbidities, smoking, and age > 65 years [2,3]. Previous studies show a significant impact of long COVID, including respiratory syndrome manifestation with declining physical function and diminished health-related quality of life, and economy [4].

The respiratory phenotype of longCOVID is one of the most common among COVID-19 survivors. Common symptoms found in the respiratory system are dyspnoea and cough, followed by anosmia and chest pain [5,6]. Various literature explain that the occurrence of these symptoms may be due to cytokine storms, lung parenchymal sequelae, cardiovascular dysfunction, hypersensitivity, and immune dysregulation [1,5,6].

Indonesia Society of Respirology (ISR), in its clinical practice guidelines, introduces post-COVID-19 Respiratory Syndrome. It is a phenotype of patients with lung symptoms or disorders that persist for more than ≥ 4 weeks. Post-COVID-19 Respiratory Syndrome is further divided into post-acute Covid-19 syndrome (≥ 4 weeks) and Chronic Post-COVID-19 (≥ 12 weeks). Symptoms included in this phenotype are cough, shortness of breath, activity limitation, chest pain, and sore or itchy throat. Patient conditions that support this phenotype are one or both of the symptoms and radiological abnormalities (pulmonary fibrosis, residual ground glass opacification, interstitial thickening, traction bronchiectasis, honeycombing) [7].

Despite the clinical burden of PCRS, little is known about early markers measurable at the time of hospital admission, which could help clinicians stratify risk and guide follow-up care. Our study collected vital signs and laboratory data from a population of COVID-19 survivors with post-COVID-19 respiratory syndrome (PCRS) and a population of COVID-19 survivors without post-COVID-19 respiratory syndrome (non-PCRS). The data collection was conducted at the time of admission. Our study aimed to find out which laboratory findings were significantly different at the time of admission between the two populations that could be markers associated with Post-COVID Respiratory syndrome.

## Methods

We conducted a case-control study by reviewing the medical records of patients with a history of hospitalization due to COVID-19 at Persahabatan National Referral General Hospital, Jakarta, in the period of 2020−2021. The minimum sample size was estimated at 49 using the single proportion formula. We then reviewed the hospital database of COVID-19 survivors admitted to Persahabatan National Referral General Hospital (2020–2021) and, after applying case–control criteria, accounting for mortality, and refusal to participate, we identified 85 eligible subjects (43 cases, 42 controls) for analysis.

Data collection was conducted from 20 December 2023–13 December 2024. Since the participants were no longer receiving treatment during the study period (they were treated in 2020–2021), informed consent was obtained by an electronic message sent to the phone numbers listed in their medical records. The study description was sent via electronic message, and participants gave written confirmation of their explicit agreement or refusal to participate in the same medium. All participants in this study were adults and provided informed consent to participate.

The study had passed an ethical review based on the Letter of Approval of the Research and Health Ethics Committee of the Faculty of Medicine, University of Indonesia, and the Dr. Cipto Mangunkusumo National Central General Hospital (FKUI-RSCM), Jakarta: KET-872/UN2.F1/ETIK/PPM.002.02/2023, and Persahabatan National Respiratory Hospital, Jakarta: 98/KEPK-RSUPP/06/2023. No patients or public were involved in this study methodology.

The data collected from medical records were demographic (age, sex, body mass index (BMI), year of admission), vital signs, and laboratory parameters. A complete-case analysis approach was applied, whereby subjects with missing data were excluded from the respective analyses. Numerical data were tested for normality using the Kolmogorov-Smirnov test to determine whether to use a parametric or nonparametric test. Categorical data are presented with the number (%), numerical parametric data are presented with the mean ± SD, and numerical non-parametric data are presented with the median (IQR). The difference is significant if p < 0.05 is obtained. Data that differ significantly are then subjected to correlation tests. Potential confounders included age, sex, and body mass index, given their established associations with long COVID. Based on Indonesian Society of Respirology recommendations, oxygen saturation (SpO₂) and respiratory rate (tachypnoea) were selected as markers of disease severity and were evaluated separately and jointly in adjusted multivariable logistic regression models. This approach allowed assessment of the robustness of associations across clinically relevant definitions of disease severity while controlling for confounding.

Considering the limitations of Indonesia’s healthcare facilities in managing COVID-19, and the fact that not all patients can be hospitalized until day 28, we adapt the ISR criteria for Post-COVID19-Respiratory Syndrome and assume the subjects in our study as cases (PCRS) and controls (non-PCRS) based on the following criteria:

Case Criteria: A subject qualifies as a case if they meet at least one of the following conditions:

1. Hospitalization for 28 days or more.
2. Hospitalization for less than 28 days with radiological results indicating fibrosis and/or the final series of radiological assessments showing either deterioration or stagnation, accompanied by one or more clinical symptoms (cough, dyspnoea, fatigue, activity limitation, chest pain, throat pain) still present at discharge.

Control Criteria: A subject qualifies as a control if they meet the following criteria:

1. Hospitalization for less than 28 days, and
2. Radiological series results show improvement or normal findings.

Notably, no participants in the present cohort experienced hospitalization lasting ≥28 days. Consequently, case classification was based on radiological findings and persistent symptoms, and the hospitalization-duration criterion did not influence group assignment. As removal of this criterion would not alter case–control status, a sensitivity analysis excluding hospitalization duration was not performed.

## Results

Table 1 shows the demographic data of the subjects in our study. We found a mean difference in age, which is older in the population case with 51.23 ± 13.388 compared to the control population 47.33 ± 13.094, but not significant (p: 0.178). Males had a higher ratio in the case group (3.78 vs 2.23), but the difference was not significant (p: 0.292). The year of admission did not show any significant trend or difference between the two populations. Mean BMI was found to be similar in both populations (26.30 (23.59–28.53) vs 26.81(24.05–34.19), p: 0.401). No significant difference was found in demographic data, indicating homogenization of the case and control population in terms of age, sex, year of admission, and BMI.

**Table 1:** Demographic differences in COVID-19 survivors with (case) and without (control) post COVID-19 respiratory syndrome.

	Case	Control	p-Value
Age	51.23 ± 13.388	47.33 ± 13.094	0.178*
Sex	M: 34, F: 9 Rasio M/F: 3.78	M:29, F: 13 Rasio M/F: 2.23	0.292**
Year of Admission	2020: 21, 2021: 22	2020: 18, 2021:24	0.580**
BMI	26.30 (23,59−28,53)	26,81 (24,05-34,19)	0,401***

*T-Test, **Chi-Square, ***Mann-Whitney U.

In Table 2, we performed a difference test on the vital signs and a correlation test on the vital signs with significant differences. Vital signs only show a significant difference in respiratory rate (case vs control, median (IQR); 24 (20–28) vs 21.5 (20–24)). The case population had a moderate correlation strength (r: 0.337) to the increase in respiratory rate.

**Table 2:** Differences and correlations of vital signs.

	Case	Control	P-Value	Correlation [r]
Systole	127 (116-140)	130 (120-140)	0.509**	–
Diastole	80 (71-89)	80 (71.8-90)	0.519**	–
Heart Rate	93 (79-106)	91.5 (83-101.8)	0.888**	–
Temperature	36.5 (36.2-36.7)	36.5 (36.2-36.7)	0.841**	–
Respiratory Rate	24 (20-28)	21.5 (20-24)	0.002**	0.337
SpO2	97 (95-98)	97 (95-98)	0.687**	–

*T-test, **Mann-Whitney U, r: Spearman Correlation Test.

Table 3 shows the difference test in complete blood count. The three main blood components that are routinely examined, namely haemoglobin, leukocytes, and platelets, did not show any significant differences. However, when the leukocyte differential count was performed, a significant difference was found between the two populations, where the Neutrophil% number was higher in the case population than the control population (80.0 (74.30–87.90) vs 75.7 (65.88–81.25), p: 0.012). Eosinophil% and Lymphocyte% numbers were lower in the case population than in the control population (median (IQR) of Eosinophil%: 0.0 (0.0–0.2) vs 0.1 (0.0–0.5); Lymphocyte%: 11.4 (7.30–17.40) vs 15.15 (10.18–25.15)). Concurrent with these findings, the NLR were higher in the case population than in the control population (7.13 (4.34–11.88) vs 4.84 (2.55–7.82), p: 0.008). The case population had a weak correlation strength in increasing Neutrophil% (r: 0.275) and NLR (r: 0.291), while the control population had a weak correlation strength in increasing Eosinophil% (r: 0.224) and Lymphocyte% (r: 0.251).

**Table 3:** Differences and correlations of complete blood count examinations.

	Case	Control	P-Value	Correlation [r]
Haemoglobin (Hb)	13.7 (12.6-15.0)	13.8 (12.13-14.3)	0.386**	–
Leucocyte (Leu)	8.43 (6.10-11.03)	6.67 (5.29-8.67)	0.088**	–
Neu %	80.0 (74.30-87.90)	75.7 (65.88-81.25)	0.012**	0.275
Eos%	0.0 (0.0-0.2)	0.1 (0.0-0.5)	0.040**	−0,224
Bas%	0.1 (0.1-0.2)	0.1 (0.0-0.2)	0.574**	–
Lym%	11.4 (7.30-17.40)	15.15 (10.18-25.15)	0.022**	−0,251
Monocyte %	6.672 ± 3.338	7.793 ± 3.704	0.146*	–
NLR	7.13 (4.34-11.88)	4.84 (2.55-7.82)	0.008**	0.291
Thrombocyte (AT)	273 (194-337)	232 (185-280)	0.149**	–

*T-test, **Mann-Whitney U, r: Spearman Correlation Test.

Clinical chemistry examination to see liver function and kidney function, and electrolytes were also examined in this study. Table 4 shows no significant difference in SGOT and SGPT, while in kidney function, there was a significant difference in BUN (15.42 (12.15–22.90) vs 11.21 (9.58–15.99), p: 0.002). The case population had a moderate correlation strength for the increase in BUN numbers (r: 0.345). In electrolyte examination, a significant difference was also found in potassium (4.1 (3.8–4.5) vs 3.75 (3.4–4.0), p: 0.000), with the case population having a moderate correlation strength for the increase in potassium (r: 0.381).

**Table 4:** Differences and correlations of liver function, kidney function, and electrolyte examinations.

	Case	Control	P-Value	Correlation [r]
Clinical Chemistry				
SGOT	44 (31-60)	47 (24-88)	0.428**	–
SGPT	55 (31-80)	43 (27.5-100.5)	0.975**	–
BUN	15.42 (12.15-22.90)	11.21 (9.58-15.99)	0.002**	0.345
Creatinine	0.9 (0.8-1.1)	0.9 (0.7–1.025)	0.161**	–
eGFR	91 (72-102)	93 (77-108)	0.434**	–
Electrolyte				
Potassium (K)	4.1 (3.8-4.5)	3.75 (3.4-4.0)	0.000**	0.381
Sodium (Na)	133.77 ± 4.602	134.76 ± 3.153	0.250*	–
Chloride (Cl)	99.577 ± 4.614	100.905 ± 4.041	0.162*	–

*T-test, **Mann-Whitney U, r: Spearman Correlation Test.

Table 5 shows the blood gas analysis examination; there was no significant difference between the case and control populations. Both populations showed mild respiratory alkalosis with slight metabolic acidosis. The control population showed more respiratory alkalosis (pH: 7.407 ± 0.48 vs 7.42 ± 0.36, p: 0.148; pCO2: 34.495 ± 6.369 vs 32.736 ± 5.399, p: 0.174; HCO3: 21.965 ± 3.867 vs 21.312 ± 2.590, p: 0.364) and metabolic acidosis (Base Excess: −2.563 ± 4.393 vs −3.326 ± 2.603, p: 0.334). However, both populations had mild disorders. D-dimer and CRP were also not significantly different between the case and control populations. D-dimer and CRP were both elevated in both populations, with a higher median in the case population but not statistically significant (D-dimer: 660 (370–1850) vs 590 (360–1025), p: 0.424; CRP: 80 (51.90–158.70) vs 73 (13.9–103.98), p: 0.116).

**Table 5:** Differences and correlations of blood gas analysis, d-dimer, and crp.

	Case	Control	P-Value	Correlation [r]
Blood Gas Analyses				
Base Excess	−2.563 ± 4.393	−3.326 ± 2.603	0.334*	–
HCO3	21.965 ± 3.867	21.312 ± 2.590	0.364*	–
O2 Saturation	97.8 (95.90-99.30)	98.05 (95.72-99.3)	0.812**	–
pCO2	34.495 ± 6.369	32.736 ± 5.399	0.174*	–
pH	7.407 ± 0.48	7.42 ± 0.36	0.148*	–
pO2	98.6 (77.50-132.50)	97.8 (78.43-143.63)	0.792**	–
Standard HCO3	23.153 ± 2.99	22.986 ± 1.89	0.758*	–
Total CO2	23.233 ± 4.252	22.571 ± 3.239	0.423*	–
Other				
D-dimer	660 (370-1850)	590 (360-1025)	0.424**	–
CRP	80 (51.90-158.70)	73 (13.9-103.98)	0.116**	–

*T-test, **Mann-Whitney U, r: Spearman Correlation Test.

Table 6 shows that, after following Benjamini–Hochberg correction, respiratory rate (p = 0.037 global; p = 0.012 clustered), blood urea nitrogen (BUN; p = 0.037 global; p = 0.006 clustered), and potassium (p < 0.001 for both) remained statistically significant. In contrast, neutrophil percentage (p = 0.089; p = 0.054), eosinophil percentage (p = 0.201; p = 0.090), lymphocyte percentage (p = 0.137; p = 0.066), neutrophil-to-lymphocyte ratio (p = 0.074; p = 0.054), and estimated glomerular filtration rate (p = 0.201; p = 0.065) were not significant after correction. These findings indicate that respiratory rate, BUN, and potassium were the most robust variables after controlling for false discovery.

**Table 6:** Benjamini–Hochberg correction applied to variables with significant p-values.

	P-Value	BH-Adjusted P-Value (Global/ Clustered)
Respiratory Rate	0.002	0.037/ 0.012
Neu %	0.012	0.089/ 0.054
Eos %	0.04	0.201/ 0.09
Lym %	0.022	0.137/ 0.066
NLR	0.008	0.074/ 0.054
BUN	0.002	0.037/ 0.006
eGFR	0.0434	0.201/ 0.065
K	<0.001	<0.001/ < 0.001

BH = Benjamini–Hochberg; NLR = neutrophil-to-lymphocyte ratio; BUN = blood urea nitrogen; eGFR = estimated glomerular filtration rate.

In models incorporating SpO₂ as the marker of disease severity (Models 1–3), potassium was independently associated with case status after adjustment for age, sex, and body mass index (OR = 4.83, 95% CI 1.66–14.10; *p* = 0.004). Neutrophil-to-lymphocyte ratio (NLR) demonstrated a modest independent association in one model (OR = 1.10, 95% CI 1.01–1.20; *p* = 0.032), whereas blood urea nitrogen (BUN) did not retain statistical significance after adjustment. These models demonstrated modest explanatory power and adequate calibration based on Hosmer–Lemeshow testing (Table 7).

**Table 7:** Comparison of multivariable logistic regression models for case population.

Model	Additional Markers Beyond Core Variables*	Significant Markers (*p* < 0.05), OR (95% CI)	Nagelkerke R²	Hosmer–Lemeshow p	Overall Accuracy (%)
1	SpO2, NLR	NLR (*p* = 0.032; OR = 1.10, 95% CI 1.01–1.20)	0.190	0.168	64.6
2	SpO2, Potassium	Potassium (*p* = 0.004; OR = 4.83, 95% CI 1.66–14.10)	0.262	0.763	65.9
3	SpO2, BUN	None	0.194	0.814	62.2
4	Respiratory rate, NLR	Respiratory rate (*p* = 0.027; OR = 1.14, 95% CI 1.01–1.28)	0.269	0.897	65.9
5	Respiratory rate, potassium	Respiratory rate (*p* = 0.016; OR = 1.13, 95% CI 1.02–1.25); Potassium (*p* = 0.009; OR = 4.47, 95% CI 1.46–13.72)	0.345	0.072	74.4
6	Respiratory rate, BUN	Respiratory rate (*p* = 0.009; OR = 1.15, 95% CI 1.04–1.29)	0.309	0.046	67.1
7	Respiratory rate, SpO₂, NLR	Respiratory rate (*p* = 0.030; OR = 1.15, 95% CI 1.01–1.30)	0.271	0.570	64.6
8	Respiratory rate, SpO₂, potassium	Respiratory rate (*p* = 0.020; OR = 1.15, 95% CI 1.02–1.29); Potassium (*p* = 0.008; OR = 4.61, 95% CI 1.49–14.26)	0.348	0.474	72.0
9	Respiratory rate, SpO₂, BUN	Respiratory rate (*p* = 0.011; OR = 1.18, 95% CI 1.04–1.33)	0.315	0.170	70.7

All models were adjusted for body mass index, sex, and age on admission. OR = odds ratio; CI = confidence interval. Core variables were included in all models. Hosmer–Lemeshow p > 0.05 indicates adequate model fit.

When respiratory rate was used as the marker of disease severity (Models 4–6), respiratory rate consistently remained independently associated with case status across all models (OR range: 1.13–1.15; *p* ≤ 0.027). The addition of potassium (Model 5) resulted in both respiratory rate and potassium remaining independently associated with case status (potassium OR = 4.47, 95% CI 1.46–13.72; *p* = 0.009) and was associated with greater explanatory power compared with models including NLR or BUN, which did not demonstrate additional independent associations.

In models incorporating both respiratory rate and SpO₂ as markers of disease severity (Models 7–9), respiratory rate remained independently associated with case status across all models (OR range: 1.15–1.18). Potassium again demonstrated a strong independent association when included (Model 8; OR = 4.61, 95% CI 1.49–14.26; *p* = 0.008), whereas NLR and BUN did not retain statistical significance. Overall, goodness-of-fit testing suggested acceptable calibration across most model specifications.

## Discussion

Our study compared demographic data such as age, gender, year of admission, and BMI, and vital signs data. In addition, our study also compared laboratory data, including complete blood count, liver function, kidney function, electrolytes, blood gas analysis, D-dimer, and CRP in the population of COVID-19 survivors with post-COVID respiratory syndrome (cases) and without post-COVID respiratory syndrome (controls).

Older age indicates a higher risk of hospitalization, mortality, need for ICU care, and more extensive lung damage on radiological images. These risks may be attributed to immunosenescence, chronic inflammation, and underlying comorbidities. Previous studies have also shown the role of the female gender, which has a higher risk of post-COVID conditions, and BMI in the obesity category, which is an independent risk factor for post-COVID conditions [8–10]. On the other hand, long-COVID conditions did not differ significantly in various COVID-19 variants [11]. The case and control populations in our study showed homogeneity, where no significant differences were found in age, gender, year of admission, and BMI in both populations. These findings may reduce the confounding of the demographics in the analysis of differences in vital signs and laboratory data in our study.

### NLR

In unadjusted analyses, our study found that the case population had higher neutrophil levels (80.0 (74.30–87.90) vs 75.7 (65.88–81.25), p: 0.012), lower eosinophils (0.0 (0.0–0.2) vs 0.1 (0.0–0.5), p: 0.040), and lower lymphocytes (11.4 (7.30–17.40) vs 15.15 (10.18–25.15), p: 0.022) in complete blood count. These differential counts resulted in a higher NLR in the case group compared to the control group (7.13 (4.34–11.88) vs 4.84 (2.55–7.82), p: 0.008). Previous studies show an association between NLR and neutrophilia with poor prognosis and severe COVID-19 [12,13]. COVID-19 patients admitted to the ICU had the lowest lymphocyte count, the highest neutrophil count, and the NLR, which shows an association between NLR and disease severity [14]. This pattern is consistent with prior studies linking neutrophilia, lymphopenia, and elevated NLR to severe COVID-19 and poor clinical outcomes [15].

However, after correction for multiple comparisons using the Benjamini–Hochberg procedure, the associations for neutrophil percentage, lymphocyte percentage, eosinophil percentage, and NLR were attenuated, suggesting that these hematologic differences may not represent robust independent signals when accounting for multiple testing. The observed unadjusted patterns remain biologically plausible, as previous literature shows severe COVID-19 is characterized by dysregulated neutrophil activation, including excessive neutrophil extracellular trap formation and impaired resolution of inflammation, which may contribute to persistent symptoms [12,16]. Additionally, eosinopenia has been described as a marker of more severe acute disease, while more severe disease is associated with persistent dyspnoea and fatigue in previous studies [17,18].

In adjusted multivariable analyses controlling for age, sex, body mass index, and markers of disease severity, NLR demonstrated a modest independent association in models incorporating SpO₂ (OR = 1.10, 95% CI 1.01–1.20; *p* = 0.032). However, this association was attenuated when respiratory rate was included. suggesting that NLR may primarily reflect underlying disease severity rather than a standalone predictor.

### BUN and potassium

On kidney function examination, BUN (15.42 (12.15–22.90) vs 11.21 (9.58–15.99), p: 0.002) and Potassium (4.1 (3.8–4.5) vs 3.75 (3.4–4.0), p: 0.000) were significantly higher in cases with moderate correlation strength (BUN r: 0.345, Potassium r: 0.381). After correction for multiple comparisons using the Benjamini–Hochberg procedure, the associations for both BUN and potassium remained statistically significant, with potassium demonstrating the strongest adjusted signal. These findings indicate that renal-related laboratory abnormalities are robustly associated with the case.

One probable explanation is the correlation between pneumonia and a high BUN level. Numerous studies indicate that a high BUN level is a sign of pneumonia severity, even utilized as one of the elements in the CURB-65 scoring system [19,20]. BUN was discovered to be both a predictor and a risk factor for COVID-19 patients’ 28-day death. A greater risk of 28-day mortality was associated with elevated BUN levels (≥7.37 mmol/L) [21]. Electrolyte abnormalities, such as hypokalemia, are typical in severe illnesses and can also happen to COVID-19 patients. Conversely, critically ill patients are prone to developing hyperkalemia. Potassium levels above 4.1 mmol/L were significantly associated with a higher risk of death, whereas lower electrolyte levels showed no such association [22].

In adjusted multivariable analyses controlling for age, sex, body mass index, and markers of disease severity, potassium demonstrated a consistent independent association with case status across several models, particularly those incorporating respiratory rate as a marker of disease severity. In contrast, BUN did not retain an independent association after adjustment, suggesting that its effect may be largely mediated by overall disease severity. Together, these findings suggest that while both BUN and potassium reflect acute illness burden, potassium may represent a more stable independent laboratory marker, whereas BUN primarily functions as an indicator of systemic and respiratory disease severity.

SARS-CoV-2–induced dysregulation of the renin–angiotensin–aldosterone system (RAAS) has been implicated in both acute COVID-19 and persistent post-COVID syndromes, reflecting altered vascular, neurohormonal, and metabolic regulation. ACE2 down-regulation following viral entry may contribute to RAAS imbalance, and long COVID has also been associated with autonomic dysfunction and ongoing low-grade inflammation, all of which are linked to systemic homeostatic control and may influence renal and electrolyte regulation. However, direct evidence demonstrating persistent or chronic potassium imbalance as a distinct pathological feature of long COVID remains limited; thus, the observed associations may reflect downstream effects of prolonged systemic dysregulation rather than a primary electrolyte disorder [23–25].

### Blood gas analyses

Blood gas studies showed no significant difference between the case and control groups. Both populations showed mild respiratory alkalosis with slight metabolic acidosis. The control population showed more respiratory alkalosis (Case vs control; pH: 7.407 ± 0.48 vs 7.42 ± 0.36, p: 0.148; pCO2: 34.495 ± 6.369 vs 32.736 ± 5.399, p: 0.174; HCO3: 21.965 ± 3.867 vs 21.312 ± 2.590, p: 0.364) and metabolic acidosis (Base Excess: −2.563 ± 4.393 vs −3.326 ± 2.603, p: 0.334). These findings are common in the COVID-19 population. Respiratory alkalosis was linked to severe COVID-19 outcomes. But for unknown reasons. In general, hypoxic stimulation causes hyperventilation in an attempt to repair hypoxia at the cost of CO2 loss in pulmonary disorders [26]. Some COVID-19 patients may not initially display substantial hypoxemia, but if respiratory alkalosis arises, they may already have compensatory hyperventilation and decline rapidly [27].

In metabolic acidosis, the amount of carbon dioxide in the lungs decreases, and breathing becomes faster. A degree of respiratory alkalosis can therefore compensate for metabolic acidosis [28]. Our study shows a significantly higher respiratory rate in the case population (case vs control, median (IQR); 24 (20−28) vs 21.5 (20−24), p: 0,002), with the case population being moderately correlated to the increased respiratory rate (r: 0.337), thus may be compensatory hyperventilation. This association remained statistically significant after Benjamini–Hochberg correction, supporting the robustness of respiratory rate as a univariate marker. In adjusted multivariable analyses controlling for age, sex, body mass index, and additional laboratory variables, respiratory rate consistently demonstrated an independent association with case status across multiple models, including those incorporating other markers of disease severity. Models including respiratory rate showed improved explanatory power compared with those without it, and respiratory rate retained significance even when combined with SpO₂, underscoring its importance as a clinical marker. Unlike several laboratory markers whose associations attenuated after adjustment, respiratory rate emerged as a stable clinical indicator linked to case status, supporting its relevance in assessing post-COVID respiratory sequelae. These findings are consistent with prior reports indicating that respiratory rates exceeding 22 breaths per minute are associated with increased mortality among hospitalized COVID-19 patients [29].

### D-dimer and CRP

This study found no significant difference in D-dimer and CRP between the case and control populations. D-dimer and CRP were both elevated in both populations, with a higher median in the case population but not statistically significant (D-dimer: 660 (370–1850) vs 590 (360–1025), p: 0.424; CRP: 80 (51.90–158.70) vs 73 (13.9–103.98), p: 0.116). Prior research has demonstrated that CRP and D-dimer are involved in the prediction of persistent COVID-19. Because of the hypercoagulable condition linked to COVID-19, elevated D-dimer levels are frequently observed in critically ill individuals. The underlying pro-thrombotic mechanism is thought to be the cause of this condition. Conversely, CRP levels have been linked to the severity of COVID-19, and ongoing CRP monitoring has been proposed as a means of forecasting a patient’s clinical trajectory. However, since CRP is a nonspecific marker influenced by various factors unrelated to COVID-19, its prognostic use requires considering baseline levels unaffected by other conditions [30,31]. Although both d-dimer and CRP were found to be elevated in our study, the nonsignificant differences in both populations may be due to the examinations performed at the time of admission. Serial studies of D-dimer and CRP may be needed to further understand the role of D-dimer and CRP in cases of post-COVID 19 respiratory syndrome [22].

### Study limitation

This study has several limitations. The relatively small sample size may limit statistical power and the precision of effect estimates. Its retrospective design restricts causal inference and is subject to information bias inherent to medical record–based analyses. In addition, misclassification of post-COVID-19 respiratory syndrome cannot be fully excluded, as case definitions relied on clinical and radiological findings at discharge. Measures of initial COVID-19 severity—such as requirements for supplemental oxygen or intensive care unit admission—were not available for inclusion in the analyses; therefore, some observed laboratory differences may reflect underlying disease severity during acute infection rather than independent risk factors for post-COVID-19 respiratory syndrome. Finally, the evaluation of multiple laboratory variables introduces the potential for false-positive findings, which was addressed using Benjamini–Hochberg false discovery rate correction.

## Conclusion

Our study identified several clinical and laboratory parameters at admission that were associated with post-COVID-19 respiratory syndrome. In unadjusted analyses, higher neutrophil levels, neutrophil-to-lymphocyte ratio (NLR), blood urea nitrogen (BUN), potassium, and respiratory rate were significantly associated with the case population; however, after correction for multiple comparisons, potassium, BUN, and respiratory rate demonstrated the most robust associations. In multivariable analyses adjusted for age, sex, body mass index, and markers of disease severity, respiratory rate and potassium consistently retained independent associations with case population, whereas associations for NLR and BUN were attenuated, suggesting mediation by overall disease severity. D-dimer and C-reactive protein levels were elevated in both groups but did not differ significantly, possibly reflecting the timing of measurement at hospital admission. While these findings support associations between early clinical and laboratory abnormalities and post-COVID-19 respiratory syndrome, further studies with larger sample sizes and serial measurements are needed to clarify their prognostic value. Early recognition of patients with persistent physiological and metabolic disturbances may help improve the monitoring and management of post-COVID-19 respiratory complications.

## Supporting information

S1 Data Dataset. Anonymized dataset.
(XLSX)
